# Cervical Proprioception Assessed through Targeted Head Repositioning: Validation of a Clinical Test Based on Optoelectronic Measures

**DOI:** 10.3390/brainsci13040604

**Published:** 2023-04-02

**Authors:** Valeria Cerina, Luigi Tesio, Chiara Malloggi, Viviana Rota, Antonio Caronni, Stefano Scarano

**Affiliations:** 1 IRCCS, Istituto Auxologico Italiano, Department of Neurorehabilitation Sciences, Ospedale San Luca, 20149 Milan, Italy; 2 Department of Biomedical Sciences for Health, Università degli Studi di Milano, 20133 Milan, Italy

**Keywords:** kinesthesia, neck proprioception, head repositioning accuracy, neurophysiology, motor control, optoelectronics

## Abstract

Neck proprioception is commonly assessed with head repositioning tests. In such a test, an operator rotates the head of a blindfolded individual to a target position. After returning to the rest position, the participant actively repositions the head to the target. Joint Position Error (JPE) is the angular difference between the target angle (however oriented in a 3D space) and the actively reached positions (the smaller the difference, the better the proprioception). This study aimed to validate a head-to-target (HTT) repositioning test using an optoelectronic system for also measuring the components of the JPE in the horizontal, frontal, and sagittal planes. The head movements requested by the operator consisted of 30° left-right rotations and 25° flexion-extension. The operators or subjects could not obtain these movements without modest rotations in other planes. Two operators were involved. Twenty-six healthy participants (13 women) were recruited (mean (SD): 33.4 (6.3) years). The subjects’ JPE in the requested (intended) plane of motion (JPE*int-component*) was a few degrees only and smaller for flexion-extensions than for left-right rotations (right rotation: 5.39° (5.29°); left rotation: 5.03° (4.51°), extension: 1.79° (3.94°); flexion: 0.54° (4.35°)). Participants’ average error in unintended planes was around 1° or less. Inter-operator consistency and agreement were high. The smallest detectable change, at *p* < 0.05, for JPE*int-component* ranged between 4.5° and 6.98°. This method of optoelectronic measurement in HTT repositioning tests provides results with good metric properties, fostering application to clinical studies.

## 1. Introduction

### 1.1. The Head-Neck System and Proprioception

The head-neck complex is provided with a refined proprioceptive apparatus whose function is crucial in controlling head posture and balance [1]. Proprioception is considered here as a synonym for kinesthesia, i.e., the perception of body segment positions and movements [2]. This capacity mainly relies upon the afferent signals originating from muscle spindles and skin receptors and, to a lesser extent, from receptors in capsules, ligaments, and joint facets [3]. Neck muscles (i.e., deep vertebral and occipital-vertebral neck muscles) show a high density of muscle spindles, like other muscles controlling precise movements (e.g., the periocular and the intrinsic hand muscles) [4]. Any impairment of the cervical afferent inputs, their sensorimotor integration, and impairments of the motor output to neck muscles (including spindles’ fibres) may result in an alteration of neck position sense [5]. Balance can be challenged when this is the case, such as in cervicogenic dizziness [6,7]. Therefore, it is interesting to realise a method that allows high accuracy, precision, and reliability in testing neck proprioception.

### 1.2. Experimental Paradigms for Estimating Cervical Proprioception

Deficits in neck proprioception can be measured with the Joint Position Error (JPE) from head repositioning tests [1,8,9,10,11,12,13,14,15,16]. In these tests, usually, subjects are blindfolded and seated on a chair with back and armrests. They are asked to reproduce target angles of left-right rotation, flexion-extension, or side-bending, previously imposed by the operator [3]. Because of anatomical reasons, minor rotations in unintended planes unavoidably accompany the intended rotations on orthogonal Euclidean planes (here, horizontal, sagittal, and transverse). Therefore by “target”, here, the angle of rotation of the subject’s head generated by the operator is considered, whichever is the plane of this angle in a 3D space.

Two different paradigms can be employed. In the “return-to-neutral” (RTN) paradigm, the subject, following the operator’s requests, moves the head to a position away from the neutral one and then returns to this neutral position [12], called Neutral Head Position (NHP). In this position, the nose is aligned on the midsagittal plane, and the eyes are aligned on the horizontal plane. In RTN tests, the starting NHP is also the target position. In the “head-to-target” (HTT) paradigm, the operator gently accompanies the subject’s head to a target position, which the subject is asked to memorise. The operator then brings the subject’s head back to the NHP, and the participant is asked to reposition his/her head to the target position [9]. The JPE is the angular difference (either signed or absolute) between the target position and the position produced by the subject when aiming at the target position once he/she feels that the intended position has been attained [8]. For a comparison between different head repositioning tests, see [17].

The RTN tests seem more frequently adopted than the HTT tests [18]. However, two critical points should be highlighted regarding RTN. First, in most published RTN tests, the head movement amplitude can be different among participants, depending on a particular individual’s active range of motion in any given direction. In addition, the neutral position is likely familiar in daily life and thus easy to recall and reproduce [1,17]. By contrast, the HTT test requires higher reproducibility because the operator sets both the target and the starting neutral position. In addition, a precise target position is rarely adopted in daily life, thus adding to the difficulty of reproduction. Hence, HTT could prove to be a more sensitive neck proprioception test than RTN.

### 1.3. Instrumental Setups for Measuring Cervical Proprioception

Whichever the trial design, various devices and measuring methods have been used to assess head movements [14,15], including ultrasound-based trackers [19], electromagnetic devices [20], electrogoniometers [21], digital goniometers [22], inertial measurement units [23,24], radiography [25], video-fluoroscopy [26], head-mounted laser pointer devices [16,27], virtual reality systems [28], and optoelectronic systems [29].

According to classic anatomy, motions are referred to three orthogonal planes (frontal, sagittal, horizontal—also said transverse). In this reference frame, head rotations are never uniplanar, even when the intended movement should be [30,31,32]. In addition, multiple trajectories can be followed before a given final position is reached. As anticipated above, the complex kinematics of sub-occipital and cervical joints impose some constraints on the trajectory and the final position. If uniaxial movements are requested, unintended components, such as side-bending on the frontal plane during intended rotations, are unavoidably associated (for a classic textbook on spine biomechanics, see [33]).

Inaccuracy and loss of precision in reproducing a given position can come both from the intended and the coupled unintended movement mechanics. This may hold particularly true in neurological or orthopaedic impairments [34,35]. Therefore, the measuring method should consider the head displacement in the three-dimensional (3D) space when head movements are studied. Optoelectronic systems are expected to return measures of human motion of the highest quality [36].

In the present study, the precision and accuracy of an HTT repositioning test based on measures from an optoelectronic system were assessed in healthy subjects. The consistency and agreement between the two operators were also measured. Normative data were eventually provided, valuable as benchmarks for clinical research.

## 2. Materials and Methods

### 2.1. Participants

Healthy adults (n = 26) were recruited at IRCCS Istituto Auxologico Italiano, a National Research Hospital in Milan, Italy, as part of a project to assess HTT repositioning in neuromuscular disorders (ClinicalTrials ID NCT04712422). Inclusion criteria were age 18–50 years, the ability to understand instructions and signing the informed consent form. Exclusion criteria were a history of mild cervical injury or neck pain the month before the test. All subjects were naïve to cervical repositioning tests when recruited. Participants were tested for their ocular preference using items 9 to 12 of the Lateral Preference Inventory [37], for their manual dominance using the Edinburgh Inventory [38], and for their foot dominance using the revised Waterloo Footedness Questionnaire [39].

### 2.2. Methods

The participants were asked to perform head movements, following a procedure formerly proposed by Loudon [9]. In the present study, the movements consisted of 30° left or right head turns (i.e., head movements in the horizontal plane) and 25° flexion or extension (i.e., head movements in the sagittal plane). These requirements accounted for the higher physiological range of neck rotation on the horizontal plane compared to the sagittal plane (around 160° and 130°, respectively [40]). Each participant was asked to move repeatedly in the four directions (right and left rotations, extension, flexion) with assistance and then autonomously (see below). An optoelectronic procedure was adopted for measuring head rotations, such as in other studies for neck kinematics [29,35,40,41,42].

### 2.3. Participants’ Preparation and Calibration Setup

Participants sat in front of a custom-made, transparent plastic panel (145 × 1 × 215 cm), on which a black grid (1.8 × 1.8 cm squares, 0.2 cm line width) was traced. They sat with the upright trunk on a chair with back support and armrests and were asked to stick to the backrest for the whole test duration.

Participants were equipped with ten spherical self-adhesive reflective markers, 1 cm in diameter, placed on the head and upper torso. Three markers corresponded to the upper torso (sternum, left and right acromion), and seven markers were fixed on a rubber “crown”.

**Figure 1:**
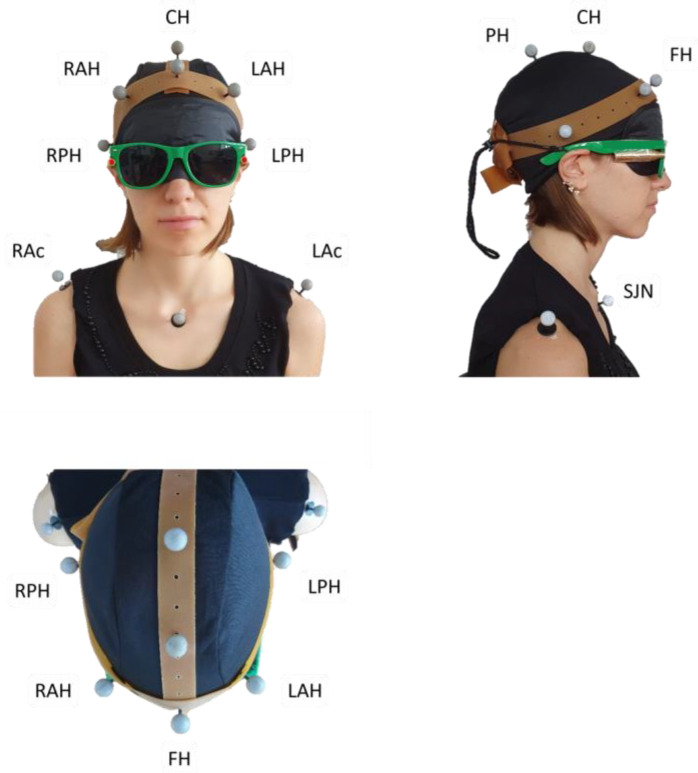
Personalised marker model (rubber “crown” set up). FH: frontal head marker; CH: central head marker; PH: posterior head marker; RAH and LAH: right and left anterior head markers; RPH and LPH: right and left posterior head markers; RAc and LAc: right and left acromion; SJN: sternum jugular notch. Two bullet-like laser pointers, projecting a red light beam, are fixed on a pair of glasses tightened posteriorly. The participant wears a sleeping mask to prevent visual cues. The subject’s gaze points downwards in the bottom panel (horizontal view) and rightwards in the right panel (sagittal view). The subject’s spatial orientation can also be inferred by looking at the corresponding marker labels.

Each participant was requested to assume a comfortable sitting posture with the head in NHP while looking straight ahead. Participants were then fit with a pair of glasses tightened posteriorly, bearing a bullet-like laser pointer on each of the two glasses’ stems, projecting a red light beam (about 0.5 cm diameter) onto the grid. A sleeping mask was applied to prevent visual clues. Figure 1 shows how the configuration of the markers and the laser pointers were arranged.

**Figure 2:**
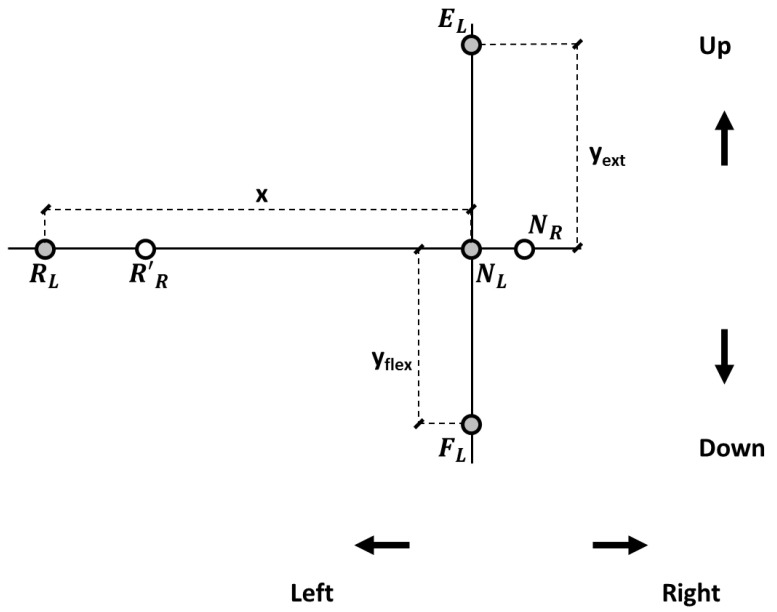
Target reference points in the panel reference frame. N*_R_* and N*_L_*: positions of the right and left lasers’ projections, respectively, with the head in the neutral position (NHP). Target reference points for the left laser pointer at 25° for neck extension (E*_L_*) and flexion (F*_L_*) and at 30° (R*_L_*) for left rotation. R’*_R_* represents the right laser pointer when rotating to the left side. An analogous procedure was followed for right rotation (not shown).

For each participant, when the head was in NHP, two target reference points were drawn where the two laser beams hit the panel (neutral left, N*_L_*, and neutral right, N*_R_*; Figure 2). The distance between the participant’s head vertex and the panel was 1 m. In the panel’s plane, the displacement of the laser beams in each direction was computed based on trigonometric relationships, and target reference points were drawn accordingly on the panel for left and right head rotations, flexion, and extension (Figure 2). Left-right head rotations corresponded for the laser spotlights to about 57 cm horizontal displacement in the panel plane. Neck flexion and extension corresponded to about 44 and 46 cm vertical displacements.

### 2.4. Experimental Design

The testing procedure started with the participant’s head held in NHP by one of two operators (a Physiatrist and a Motor and Sport Sciences specialist, both males).

A whole experimental session included 32 trials. A trial consisted of two consecutive repetitions (one assisted and one autonomous) of the same movement in one direction. Four directions were tested, i.e., right rotation, left rotation, extension, and flexion. Each of the four directions was tested four times with the first operator (i.e., four trials per direction). This implied an experimental sequence of 16 trials (thus including 16 assisted and 16 autonomous repetitions). After five minutes, to estimate the inter-operator reliability (see below), the whole sequence was repeated with the second operator, thus giving a total of 32 trials (32 assisted and 32 autonomous repetitions) during the entire experimental session. The experimental design is sketched in Supplementary Figure S1.

The operator stood behind the subject and gently placed his hands on the participant’s head during the first repetition (i.e., the assisted repetition). The subject’s head was positioned in NHP. Then, the head was gently steered to the Target Position (TP). TP was achieved when the laser beams’ projections matched the panel’s target reference points (Figure 2), ideally corresponding to 30° for right and left rotations and 25° for flexion and extension. However, the Operator could not avoid some inaccuracies (see further for the Operator Positioning Error, OPE). The TP was considered, in any case, the head position set by the operator.

Once TP was attained, the participant was asked to memorise it, and after about three seconds, the operator gently accompanied the head back to the NHP. The whole assisted motion, back and forth, took about eight seconds. Then the operator retracted his hands. After 3–5 s, the participant was asked to reach the TP autonomously (the second repetition within the trial, i.e., the autonomous repetition) at a freely chosen speed. Finally, participants held the actively Reached Position (RP) for 3–4 s before returning to the NHP. No verbal cues on the results were provided. The procedure was then repeated for the following trials.

Despite the pseudo-randomisation procedure (see below), participants could distinguish the voice of the two operators providing instructions. The participants maintained the sitting position, the same eye mask and glasses, and the arrangement of the markers for the whole experimental session. No feedback about the repositioning performance was given to the participants during testing.

The order of trials and operators was quasi-randomised as follows. The operator testing the first participant was randomly chosen, and the first direction was tested for the first participant. After the two repetitions in the first direction, the other trials followed according to a default series (i.e., neck flexion was followed by right rotation, extension, and eventually left rotation), which looped between operators and participants. An example of test randomisation is reported in Supplementary Table S1.

### 2.5. Variables and Measurements

For each trial, JPE (i.e., the repositioning error) was calculated by comparing the head’s position in RP and TP. As previously illustrated, reaching a particular position of the head involves movements that cannot be restricted to a single plane. Therefore, the JPE components projected onto the horizontal, frontal, and sagittal planes (i.e., three planar JPEs) were calculated. An intended JPE component (JPE*int-component*) and two unintended components were identified for each trial. JPE*int-component* consisted of the planar JPE projected in the same plane of the movement’s direction requested to the operator (i.e., the sagittal plane for trials testing flexion and extension and the horizontal plane for trials testing right and left rotations). The remaining two planar JPEs were dubbed *unintended* JPEs. As an example, for a trial testing right head rotation, the JPE*int-component* consisted of the planar JPE calculated on the horizontal plane. In contrast, the unintended components were the ones projected on the sagittal and frontal planes (JPE*sagittal* and JPE*frontal*, respectively).

**Figure 3:**
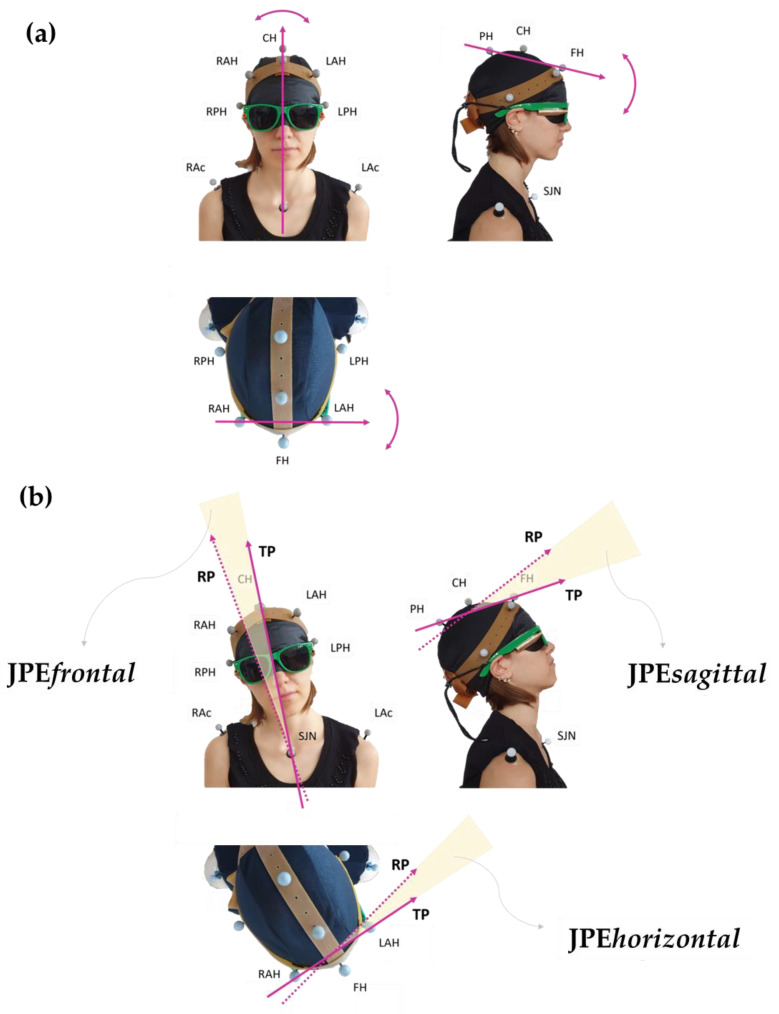
Panel (**a**) shows the vectors chosen to assess the head’s positions in the three classic anatomical orthogonal planes: $\vec{RAH;LAH}$ allows evaluation of the rotation movements in the horizontal plane, $\vec{PH;FH}$ for flexion-extension movements in the sagittal plane, and $\vec{SJN;CH}$ for side-bending movements in the frontal plane. Panel (**b**) provides a graphical visualisation of the planar JPEs calculated with respect to each plane: TP and RP refer to the vector “fixed” in its coordinates at the Target Position (TP; the one set by the operator during the assisted movement) and at the Reached Position (RP; the one attained during the actively performed movement), respectively. In this representation, examples of movements are shown separately in the frontal, sagittal, and horizontal views. Subject’s spatial orientation as in Figure 1.

To calculate the three planar JPEs, with respect to the laboratory frame of reference, the following three versors were defined: $\vec{LAH;RAH}$ for the error component in the horizontal plane (angular errors in right and left rotations), $\vec{PH;FH}$ for the error component in the sagittal plane (angular errors in flexion and extension), and $\vec{SJN;CH}$ for the error component in the frontal plane (angular errors in left and right side-bending) (Figure 3).

For all trials, the JPEs were calculated as the angle between each versor at RP and that same versor at TP, according to the following formula:

(1) $$JPE=∡\left|Target\;Position;\;Reached\;Position\right|$$

Planar JPEs can be positive or negative. A 0° value was conventionally assigned to the TP. Here, a positive planar JPE*int-component* indicates that the participant exceeded the TP position (i.e., overshooting the target). In contrast, a negative sign indicates that the target was undershot. In addition, positive JPE*sagittal* or JPE*horizontal* indicate rotation to the right or extension, respectively. For JPE*sagittal* and JPE*horizontal,* a negative sign is used for left rotation and for flexion, respectively. Last, a positive JPE*frontal* indicates side-bending to the right in the frontal plane (i.e., clockwise from behind the subject, i.e., from the operator’s perspective). At the same time, a negative sign stands for side-bending to the left (i.e., counterclockwise from the operator’s perspective). Therefore, if the head positions in RP and TP were perfectly matching, the three planar JPEs (hence, the “absolute”, JPE*3D*, see below) would all be equal to zero.

In addition to the calculation of the three planar JPEs, the optoelectronic system allows direct measurements of the angular error on whichever plane the error is measured on without deriving it from the error components on the three orthogonal planes of reference. This angular error “in a 3D space” was called JPE*3D*. The value of JPE*3D* is absolute so it can only be equal to or greater than zero. For each trial, JPE*3D* was calculated as the angle between versor $\vec{PH;FH}$ at RP and $\vec{PH;FH}$ at TP. The JPE*3D* was used as a synthetic index, as it is equal to zero when the head positions in RP and TP are perfectly matching.

The operator was requested to assist with the movements of the head so that the laser projections would match the panel’s target reference points, and this position was set as TP. However, TP could not perfectly match the ideal angles (30° for rotations and 25° for flexion or extension). Therefore, the mismatch between the TP and the ideal angles during the operator-assisted repetitions was defined as OPE. OPE was calculated as the difference between the ideal angles and the angles corresponding to the three versors at TP (i.e., $\vec{RAH;LAH}$ for the horizontal plane, $\vec{PH;FH}$ for the sagittal plane, and $\vec{SJN;CH}$ for the frontal plane).

(2) OPE=∡|Aimed position; Target position|

Similarly to JPE, the analysis of OPE involved both the components projected onto the sagittal, horizontal, and frontal planes with respect to the laboratory frame of reference (i.e., planar OPEs: OPE*int-component*, OPE*sagittal*, OPE*horizontal*, and OPE*frontal*) and the OPE*3D* (i.e., the OPE of whichever plane the error is measured on, not projected onto the three classic orthogonal planes).

Of note, as previously stated, the measurement of planar JPEs (both intended and unintended) and JPE*3D* came from the comparison between RP and TP within the same trial, no matter how large OPE was and whichever its orientation.

**Figure 4:**
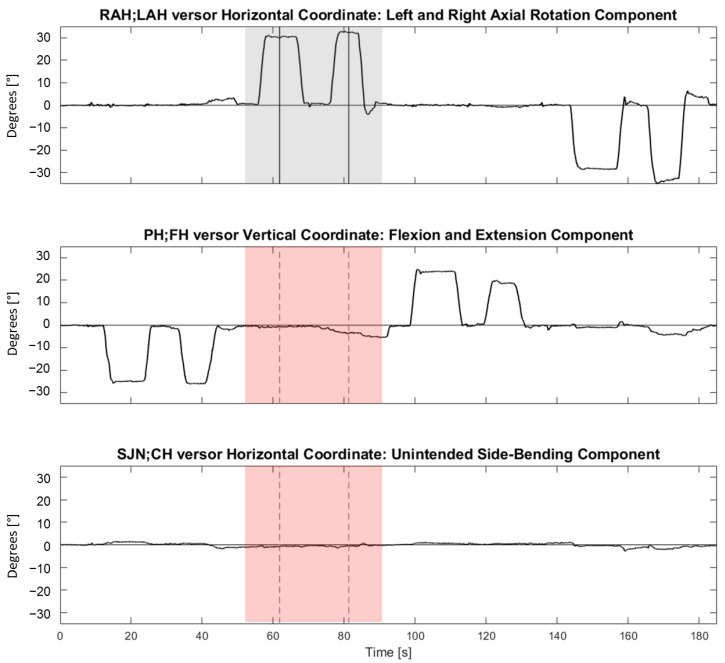
Representative synchronous tracings of the time course of the angles between $\vec{RAH;LAH}$**,** $\vec{PH;FH},$ and $\vec{SJN;CH}$ versors and the Neutral Head Position during four consecutive trials (i.e., flexion, followed by right rotation, extension, and left rotation). From top to bottom, horizontal $\vec{RAH;LAH}$ (referring to right and left neck rotations), vertical $\vec{PH;FH}$ (referring to neck flexion and extension), and horizontal $\vec{SJN;CH}$ (referring to neck right and left side-bending) coordinates are shown. On the ordinate, coordinates are expressed in angular degrees (°). On the abscissa, time is reported (in seconds). The vertical lines indicate time events manually identified during the analysis for a right rotation movement. The grey-shaded area highlights the intended component (horizontal coordinate) during a right rotation trial. The pink-shaded areas highlight the synchronous unintended components of flexion-extension and side-bending in the second and third panels from the top, respectively. For each trial, two repetitions were performed in the same direction (an operator-assisted repetition, followed by an autonomous repetition). For each repetition, a plateau in the tracings was manually identified. The TP or the RP corresponded to the mean, computed over two seconds, of the versor trace in the mid of the plateau (vertical segments). If more plateaus were present, the furthest plateau from the ideal target (for TP) or from the TP (for RP) was considered.

Figure 4 shows representative synchronous tracings of the time course of the angles between $\vec{RAH;LAH}$**,** $\vec{PH;FH}$, and $\vec{SJN;CH}$ versors and the NHP during four consecutive trials (each trial was made of two repetitions in one of the four directions).

### 2.6. Statistics

Planar JPEs, JPE*3D*, planar OPEs, and OPE*3D* were calculated for all 32 trials for each participant. Mean and standard deviation (SD) were chosen as central tendency and dispersion indices, respectively.

The statistical questions concerned are:

- The sample’s accuracy and precision in the four tested directions (i.e., JPE value);
- The dependence of these variables on operators, directions, and their interaction.

Repeated measures ANOVA were used to assess if JPEs differed according to the following factors: operator, direction, and the interaction between operator and direction. The same analysis was performed on OPEs. The mean values of the four trials in each direction were used as the response variable in the ANOVA models. The normality of data distributions was assessed using Shapiro–Wilk’s test. The applicability of the ANOVA models was checked through the normality of the residuals.

Student’s *t*-tests were used as post-hoc tests.

The inter-operator reliability was tested through Bland–Altman plots as a measure of agreement between the JPEs associated with the first and the second operator [43].

The type 1 error probability was set at 0.05. Indeed, in the present work, multiple tests of significance are performed, and applying corrections for multiple comparisons is customary in these cases, thus abating the p-level. However, one of the welcome outcomes of the study is demonstrating no between-operator differences. Therefore, we preferred a conservative approach, making achieving (unwelcome) significance easier.

### 2.7. Software, Instrumentation, and Signal Analysis

The 3D displacement of the markers was captured using eight near-infrared stroboscopic cameras (Smart-D optoelectronic system; BTS™ Bioengineering SpA, Milan, Italy, sampling rate 100 Hz). The centre of a reflective marker can be located with a mean error of 0.37 mm (SD 0.28 mm) (Thor2 calibration system, BTS SpA). Each raw marker’s signal was first interpolated through a cubic spline curve and then smoothed through a triangular window bandpass filter. Data analysis was conducted in SMART-Analyzer through a customised protocol (BTS Bioengineering Spa, Milan, Italy). Graphic representations were plotted using SigmaPlot ^TM^ (version 14.0, Systat Software Inc., San Jose, CA, USA) and MATLAB^TM^ (version R2020a, MathWorks Inc., Natick, MA, USA). The statistical analysis was performed using Stata^TM^ (version 14.0; Stata Corp., College Station, TX, USA).

## 3. Results

The demographic information of the participants is given in Table 1.

In total, 831 trials were analysed (i.e., 26 participants performing 32 trials each; data from one trial of right rotation were accidentally lost).

**Figure 5:**
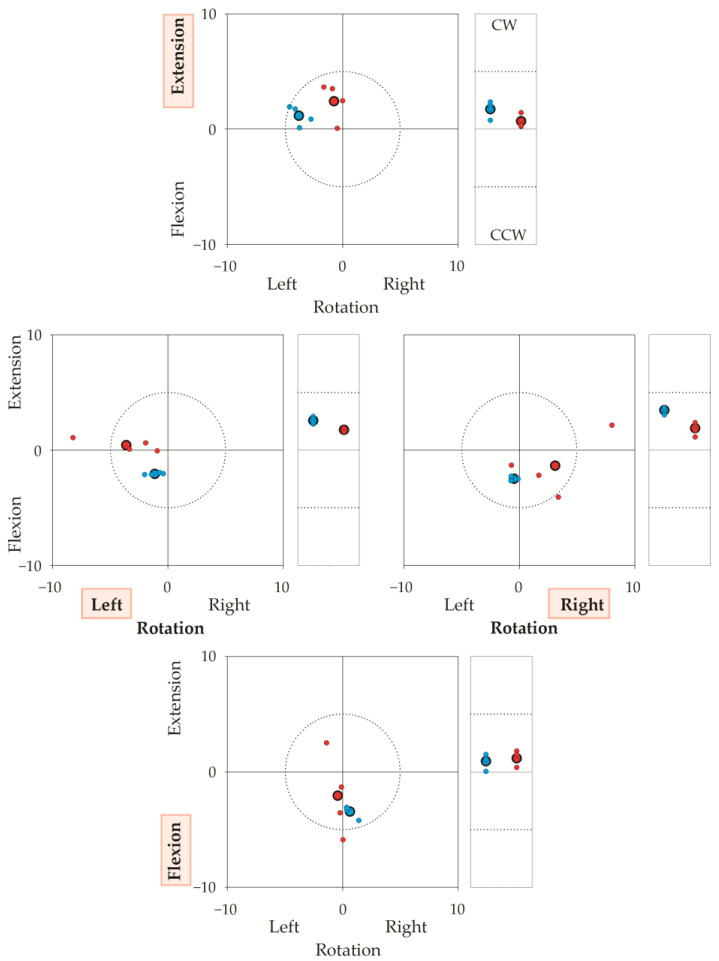
The figure illustrates the target reference points (0,0 coordinates), the Target Positions (TPs) and Reached Positions (RPs) in a representative sequence of 32 repetitions (16 trials) performed by a participant–operator couple. Axes give angular errors in degrees. TP is the head’s final position when the participant’s head was moved by the operator; RP is the position reached autonomously by the participant. Each small dot corresponds to the TP (blue) or the RP (red) of a single repetition. Big dots with a bold border represent the average of the four repetitions. In a trial, the operator first rotated the participant’s head from the neutral head position to the target reference point (30° for left or right rotation, 25° for flexion or extension). If the operator was errorless, TP coincided with the target reference point (0,0 coordinates). Then, after the operator-assisted repetition, the participant autonomously turned their head from the neutral head position to TP. If the participant was errorless, RP coincided with TP. Thus, if both the operator and the participant were errorless, RP and TP coincided with the target reference point. To stress, the operator had to be accurate and precise with respect to the target reference point, while the participant had to be accurate and precise with respect to TP. For each trial, OPE was calculated as the difference in degrees between the coordinates of the TP and 0,0. Participants completed (in quasi-random sequence) neck extensions (upper graph), left-right rotations (middle graphs), and neck flexions (lower graph). These intended directions are indicated in red shaded background. Squared graphs: TPs and RPs in the sagittal (*y*) and horizontal (*x*) planes, in degrees (°). Dotted circumferences in the square graphs encase a 5° radius centred on the target reference point. Rectangular graphs: (unintended) side-bending rotations in the frontal plane, in degrees (°). CW: clockwise from the participant’s (and the operator’s) perspective; CCW: counterclockwise. Horizontal dashed segments in the rectangular graphs mark 5° from the target reference point. It is worth stressing that JPE was the difference between RP and TP from the same trial. Take, for instance, the extension task (upper square panel). The average of the operator’s errors (big blue dots, bold border) was about 4° towards the left in rotation, 1° in extension, and 2° in CW (e.g., towards the participant’s right) side bending (top rectangular panel). Regarding the participant’s autonomous repetitions, the big red dot (average value marked with bold borders) indicates the positions reached by the participant with respect to the 0,0 coordinates, i.e., the positions “seen” by the optoelectronic system. However, the error is computed with respect to the TPs (i.e., the operator-assisted repetitions). For instance, in the top square graph, the average position shown by the participant is very close to the 0,0 coordinates, but it is rather far from the position requested by the operator. There, an error of about 3° was found.

Figure 5 illustrates a representative sequence (i.e., 16 trials) performed by one operator–participant couple, showing the coordinates of both TPs and RPs with respect to the laboratory’s frame of reference (the coordinates of the target reference point for each direction, were chosen as 0,0 coordinates). The figure highlights that both the operator’s and the participant’s accuracy and reproducibility were high. In this regard, note that the “target” was not the same for the operator-assisted repetitions and for the autonomous repetitions: operator-assisted repetitions (i.e., TPs, blue dots) had to match the target reference points (corresponding to coordinates 0,0 in the squared graphs and to coordinate 0 in the rectangular graphs of Figure 5); on the contrary, each autonomous repetition (i.e., RPs, small red dots) had to match the TP of the same trial. Regarding the operator’s accuracy, the mean TP (big blue dot with a bold border) was within 5° from the target reference point. The mean RP (big red dot with a bold border) was a few degrees from the mean TP. To note, unintended components were small in all directions.

### 3.1. Operators’ Accuracy

The statistical analysis showed that the mean reproducibility between the two operators was high. In fact, the “Operator” factor and the interaction between the “Operator” and the “Direction” factors from the repeated measures ANOVA were not significant for the OPE*int-component* nor for OPE*3D* or for the unintended OPEs (Table 2). On the contrary, the “Direction” factor resulted as significant in the model for all the variables (OPE*int-component*: F(3, 175) = 3.99; *p* = 0.009; OPE*sagittal/horizontal*: F(3, 175) = 10.87; *p* < 0.001; OPE*frontal*: F(3, 175) = 25.28; *p* < 0.001; OPE*3D*: F(3, 175) = 3.04; *p* = 0.031). The residual sum of squares of the model was 134 for OPE*int-component*, 139 for OPE*sagittal/horizontal*, 230 for OPE*frontal*, and 185 for OPE*3D*, with 175 degrees of freedom (not shown in the table).

In particular, OPE was the largest for the component of error on the frontal plane (OPE*frontal*) during right and left rotations.

### 3.2. Patients’ Accuracy

Table 3 reports the repeated measures ANOVA of the JPEs.

The “Direction” factor was significant for both JPE*int-component* and for JPE*3D* (F(3, 175) = 43.48; *p* < 0.001; F(3, 175) = 26.03; *p* < 0.001), while neither “Operator” nor the “Operator × Direction” interactions were significant.

On the contrary, the “Operator × Direction” interactions were significant for the unintended JPEs (JPE*sagittal/horizontal:* “Direction” factor: F(3, 175) = 29.91; *p* < 0.001; “Operator × Direction” interaction: F(3, 175) = 5.80; *p* < 0.001; JPE*frontal:* “Operator” factor: F(1, 175) = 17.15; *p* < 0.001; “Direction” factor: F(3, 175) = 7.15, *p* < 0.001; “Operator × Direction” interaction: F(3, 175) = 4.69, *p* = 0.004) and post-hoc testing showed differences between the two operators for both the unintended JPE*sagittal/horizontal* and JPE*frontal*.

The residual sum of squares of the model was 1191 for JPE*int-component,* 313 for JPE*sagittal/horizontal*, 226 for JPE*frontal*, and 641 for JPE*3D*, with 175 degrees of freedom (not shown in the table).

Post-hoc tests also showed that JPE*int-component* and JPE*3D* were larger for left and right rotations than for flexion and extension. These results were confirmed when the analysis was run on the JPE normalized on the amplitude of the ideal target positions in order to account for the difference in the requested movements (i.e., 30° and 25° for right-left rotations and flexion-extensions) (data not shown).

The coefficients of variation of the JPE*3D* did not differ substantially across operators and directions (Table 3, third and fifth columns from left).

**Figure 6:**
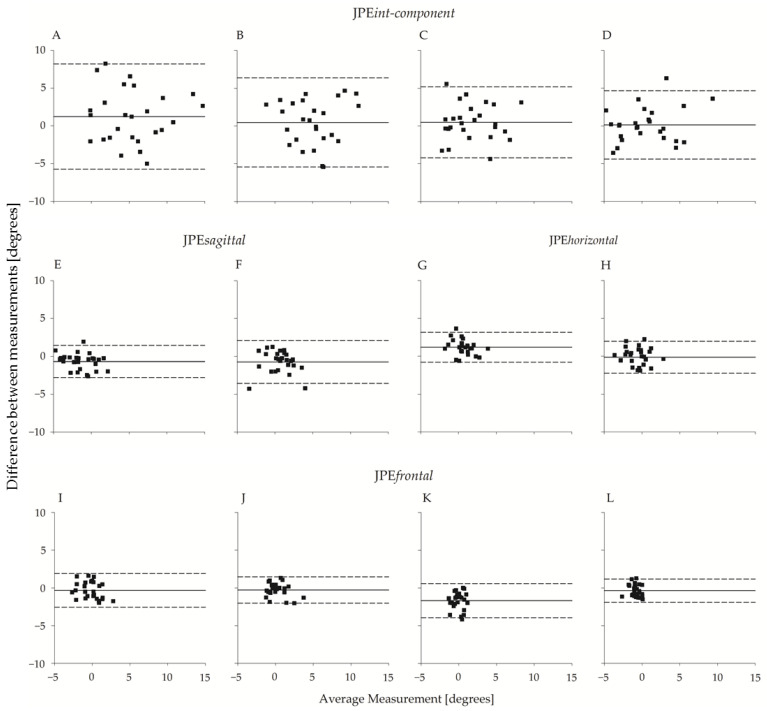
Bland–Altman plot (N = 26) of JPE*int-component*, JPE*sagittal*, JPE*horizontal*, and JPE*frontal* with respect to the two operators. From top to bottom, the first row refers to JPE*int-component*, the second row refers to JPE*sagittal* and JPE*horizontal*, and the third row refers to JPE*frontal*. In the uppermost row, each panel refers to one of the four directions: (**A**) right rotation; (**B**) left rotation; (**C**) extension; (**D**) flexion. Below (**A**,**B**), the corresponding unintended components in the sagittal and frontal planes ((**E**,**F**); (**I**,**J**), respectively) are considered. Below (**C**,**D**), the corresponding unintended components in the horizontal and frontal planes ((**G**,**H**); (**K**,**L**), respectively) are considered. Abscissa axes report between-operators average (°), and the ordinate axes report between-operators difference (°). The continuous line refers to the mean of the differences; the dashed lines represent 95% limits of agreement.

The between-operator agreement is shown in Figure 6, which reports the Bland–Altman plots of the planar JPE outcomes (JPE*int-component*, JPE*sagittal*, JPE*horizontal*, and JPE*frontal*). The operators’ bias was small, amounting to a few tenths of a degree in all four directions. Agreement between the two operators was high and slightly higher for flexion-extension movements than for left-right rotations. As an example, the 95% limits of agreement (LoA) for JPE*int-component* ranged from ~4.5° to ~4.7° and from ~5.9° to ~7° for flexion or extension and left or right rotations, respectively.

### 3.3. Additional Analyses: Hypermetric and Hypometric Movements

As shown in Figure 5 and by the Bland–Altman plots (Figure 6), participants could overshoot (e.g., extension graph in Figure 5) or undershoot the TP (e.g., flexion graph in Figure 5). This aspect is investigated in greater detail below.

**Figure 7:**
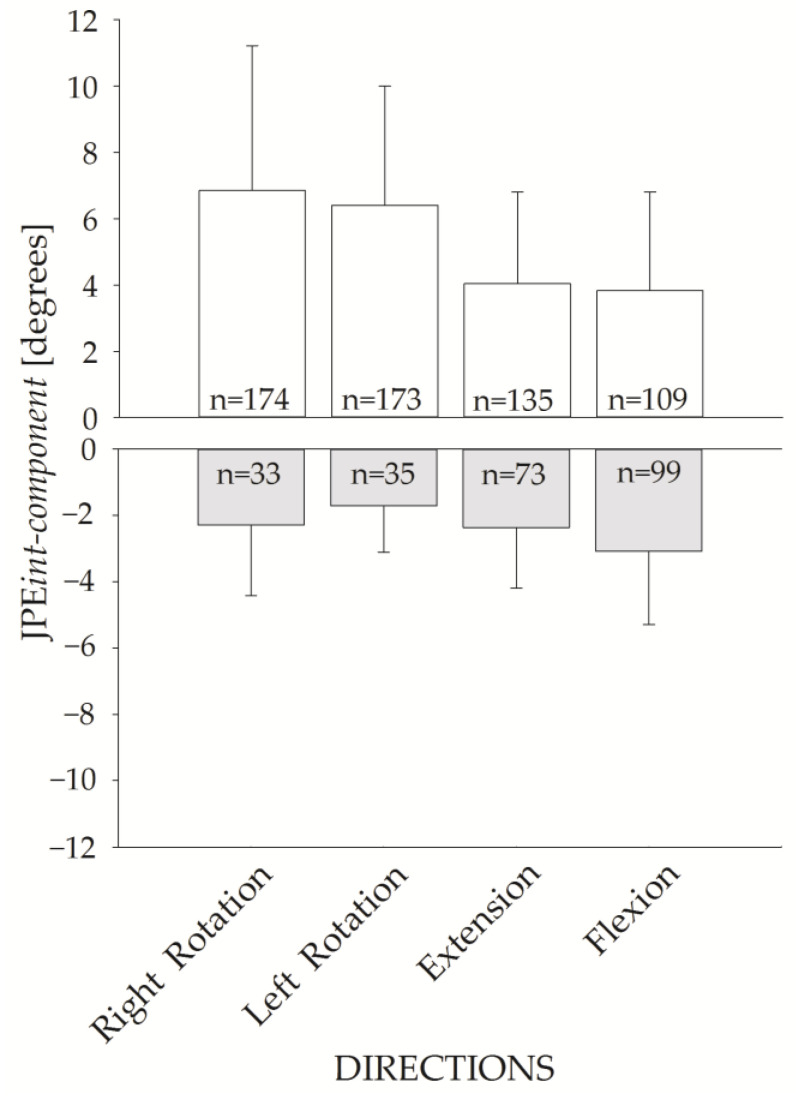
JPE*int-component* bar plot for the four movement directions. Hypermetric (white bars) and hypometric (grey bars) JPE*int-component* (mean and standard deviation) are represented. The corresponding number of hypermetric vs. hypometric trials is shown inside each bar.

Figure 7 shows the JPE*int-component* for the four requested directions. The upper panel shows the JPE*int-component* of the hypermetric trials (positive values), while the lower panel shows the values for hypometric trials (negative values). JPE*int-component* of the hypermetric trials were larger than the hypometric ones.

Of note, trials were more frequently hypermetric than hypometric (591 hypermetric vs. 240 hypometric trials).

## 4. Discussion

The current work assessed the accuracy of an HTT repositioning test for evaluating head-neck proprioception recorded with an optoelectronic system. The method of assessment of cervical proprioception presented in this study adds to the current literature, which is heterogeneous regarding experimental designs, instrumental devices, and analysis methods. 

Briefly, the present study:

- shows that the operators were accurate and precise in positioning the participants’ heads during assisted movements, a piece of information missing in other studies;
- shows that the measurement error associated with the testing procedure was low;
- provides normative data on the repositioning errors (i.e., planar JPEs and JPE*3D*);
- demonstrates that, in head repositioning, movements in unintended planes are small, at least in these healthy controls.

Thus, this HTT paradigm for assessing head-neck proprioception provides accurate, precise, and reliable results, presumably more than previous studies adopting other protocols and devices.

### 4.1. JPE Analysis

The overall findings of the present paper on JPE are close to those provided in the existing literature for healthy individuals involving other measuring devices (e.g., ultrasound devices, electromagnetic systems). More specifically, Kristjansson [20], Malmström [44], and Chen [45] reported mean JPEs for 30° right (5.0°, 6.3°, and 4.8°, respectively) and 30° left rotations (7.2°, 5.2°, and 4.8°, respectively) similar to those reported here (5.39° and 5.03°, respectively, for JPE*int-component*; 6.62° and 5.78°, respectively, for JPE*3D*). To the authors’ knowledge, the present results are the first available for HTT repositioning in 25° flexion and extension movements (0.54° and 1.79°, respectively, for JPE*int-component*; 3.77° and 4.10°, respectively, for JPE*3D*).

We found greater JPE*int-component* and JPE*3D* for right-left head rotations than for flexion or extension. Moreover, the frequency of hypermetric trials was higher in horizontal rotations, and JPE*int-component* was larger in hypermetric than in hypometric trials. Altogether, these findings point out that healthy controls commonly overshoot the target in HTT repositioning and that this overshooting seems more remarkable for movements occurring in the horizontal plane.

Malmström [46] and Armstrong [47] have already pointed out that the target is usually overshot during head repositioning tests. More broadly, overshooting the target looks like a general phenomenon in joint repositioning, as also shown in studies involving the shoulder [48], the wrist [49], and the knee [50,51].

The fact that overshooting is larger for horizontal rotations than for flexion or extension can have different explanations. First, simply because of Fitts’ law [52], a more significant overshooting would be expected if movements on the horizontal plane were faster than those on the sagittal one. However, the current work did not investigate the movement’s trajectory but only the head’s final position. Thus, this hypothesis cannot be ruled out (see study limitations). Second, one could suggest that this difference is due to the broader target angles chosen for right and left rotations than for flexions or extensions (30° and 25°, respectively). In this regard, it has also been shown that the larger the movement, the more significant the overshoot [53]. However, this seems not to be the case in the present study (see Results). Third, horizontal and vertical head movements may underlie different motor control strategies. Indeed, in daily activities, horizontal eye movements are primarily employed [54], and humans generate more horizontal saccades than vertical saccades during visual search tasks [55]. Head movements are coupled with eye movements during visual exploration, as they are typically needed to shift the visual field [56]. From this perspective, there may be differences in how horizontal and vertical head rotations are controlled and exploited for visual exploration and postural control [57].

Further, it remains to be explained why flexion showed the smallest JPE*int-component* and JPE*3D* across the four movement directions tested. Slow flexion can be obtained by relaxing the neck extensor muscles, yet “eccentric” contractions seem to involve a lower, not a higher, position sense, at least in studies on knee position sense [58].

### 4.2. Inter-Operator Reliability

In clinical practice, operators can be different between successive assessments of the same patient. Inter-operator reliability is thus a relevant parameter of the test procedure. Moreover, given the HTT testing procedure employed in the current experimental paradigm, assessing inter-operator reliability is particularly relevant: the operators were actively involved in the testing procedure by steering the motion of the participant’s head; furthermore, JPE was calculated by comparing the operator-assisted and the autonomous repetitions of the same motion. On these bases, it can be easily hypothesised that the operators, by imposing variable target positions, could add unwanted variability to measures. However, the present study’s results do not evidence statistically significant differences in the OPE outcomes (OPE*int-component*, OPE*sagittal*, OPE*horizontal*, OPE*frontal*, and OPE*3D*). Therefore, no differences in the target positions between the two operators were found. The results also highlight good consistency in the measures of the planar JPEs between two professional operators (see the Bland–Altman plots in Figure 6). In the ANOVA, significant differences between the two operators were found only for JPE*frontal*, where the largest difference was found in the extension trials (around 1.6° of difference).

In everyday clinical practice, presumably, only one operator will perform the test, and thus only one sequence (i.e., 16 trials) will be performed. For this reason, reference values for planar JPEs and for JPE*3D* for the first sequence of 16 trials performed by the 26 participants are provided in Supplementary Table S2.

### 4.3. Comparison between Optoelectronic Systems and Other Measuring Devices

This is the first study using an optoelectronic system for measuring cervical JPE in an HTT repositioning test. Optoelectronic systems are the current gold standard in movement measurement [36,59,60], and they are used to calibrate other systems, such as inertial sensors and markerless devices (i.e., Kinect™ and Augmented Reality Devices). Further, optoelectronic systems allow a complete evaluation of head motion in the 3D space, while other experimental settings may show technical difficulties in assessing specific movement directions. One additional strength of the optoelectronic system is that, contrary to measuring devices that need to be applied to the subject’s body spanning a joint (e.g., electrogoniometers), it avoids bias due to additional exteroceptive information provided to the subject.

When no significant bias is present (like in the present study, characterised by a very low systematic error [43,61], as shown in the Bland–Altman plots of Figure 6), the LoA approximate the Smallest Detectable Change (SDC), also called Minimal Real Difference. In this study, the LoA for JPE*int-component* (equal to 6.98° and 5.90° for right and left rotations, respectively) are similar to the ones reported in two previous studies assessing repositioning in the right and left rotations with an HTT paradigm involving a three-dimensional ultrasound device [15,45]: SDC was 6.10° and 8.04° for right rotation, and 4.46° and 5.27° for left rotation (SDC—at *p* < 0.05—was calculated from the standard error of measurement (SEM) provided in the articles, according to the formula $SDC=1.96\times \sqrt{2}\times SEM$).

### 4.4. Study Limitations and Suggestions for Further Research

The clinical applicability of this method remains limited. First, the procedure’s feasibility and tolerability remain to be tested on patients, such as those with a musculoskeletal neck disorder (e.g., chronic neck pain, whiplash) or a neurological disease affecting the neck (e.g., cervical dystonia, myopathies). Moreover, test-retest reliability across distinct sessions and the corresponding SDC [62] remain to be assessed. The SDC is a relevant property of any measure since it permits concluding if a single patient is “significantly changed”, e.g., due to disease progression or treatment. A twin study on these measurement properties is ongoing in our laboratories.

The sample size can be considered somewhat limited, although previous studies involving groups of healthy individuals have recruited similar numbers of participants [21,63,64,65]. Future studies will be able to provide more generalizable results by recruiting larger samples.

More refined (but more complex) methods for measuring accuracy in repositioning procedures have been developed. For example, in an RTN test, Grip et al. studied the JPE and the variation in the head rotation axis during head repositioning [35]. These authors concluded that the analysis of the axis of motion provides additional information on the proprioceptive control of neck movements.

The current analysis focused only on the head position at the repositioning end. However, if one’s aim is distinguishing patients from controls, other movement parameters, the most relevant being the 3D path of movement, movement velocity, acceleration, and jerk, should be considered. To note, the speed of head motion has many implications for head repositioning. The angular velocity (and acceleration) of the assisted head motions may affect the relative contribution of the subject’s vestibular and neck proprioceptors in sensing head position. Further, the velocity of the active motion can influence head repositioning accuracy [52]. Future research could also consider several parameters of the movement trajectory, not just the final movement position.

Optoelectronic systems suffer from some drawbacks in clinical practice (e.g., the equipment is expensive, recording is time-consuming, and high expertise in signal processing and analysis is required). Further, markers applied on the skin could lead to measurement artefacts due to the displacements of soft tissues with respect to bony landmarks [66,67]. Therefore, to increase the clinical feasibility of the HTT repositioning test evaluated here, future studies should compare the validity of cheaper and easier-to-use measurement instruments (e.g., inertial measurement units [24]).

Finally, the test evaluated here is considered a test of cervical proprioception. Nevertheless, it should be stressed that (i) blindfolded head rotation activates both neck proprioceptors *and* the vestibulum, and (ii) the test assesses both the ability to detect and memorise the target position *and* the motor ability. Both sensory *and* motor impairments could lead to a significant repositioning error. Regarding the first point, it is worth noting that even if the effect of vestibular feedback and cervical proprioception are combined in HTT testing, the vestibulum is preferentially activated by head rotation with high acceleration [68], such as in “as fast as possible” movements, which were not tested here. Regarding the second point, sensory feedback is critical for the success of accurate movements performed at low speeds. On the contrary, strong muscular contractions, during both muscle shortening and lengthening, are essential for fast, ballistic movements [69].

## 5. Conclusions

HTT repositioning should be considered a test of cervical proprioception useful in clinical practice. Optoelectronic measurements ensure high accuracy, precision, and reliability. Therefore, the results of the present study lend themselves to providing reference standards for clinical applications based on simpler technologies. The movements tested in this paradigm are similar to those trained in the neck proprioceptive exercises, emphasizing movement accuracy and precision (rather than speed or force) and reliance on proprioception (which can even be augmented through biofeedback devices, see [70]).

## Figures and Tables

**Table 1:** Participants’ demographic data (N = 26).

Gender, Female/Male	13/13
Age, mean (SD), yr	33.4 (6.3)
Height, mean (SD), cm	173.9 (9.1)
Weight, mean (SD), kg	71 (13.1)
Ocular preference, right/both/left	14/7/5
Dominant upper limb, right/left	25/1
Dominant lower limb, right/left	25/1

**Table 2:** (a) Mean (standard deviation) in degrees (°) and ANOVA results for Operator Position Error (OPE) outcomes. (b) Post hoc Student’s *t*-test on contrasts between pairs for OPE*int-component* and OPE*3D* for the “Direction” factor. The *p*-value was set at *p* < 0.05.

**(a)**					
Movement	Operator 1	Operator 2	*p*-Value (Operator)	*p*-Value (Direction)	*p*-Value (Operator × Direction)
OPE*int-component*			0.673	0.009 *	0.182
Right Rotation	1.29 (0.96)	1.28 (1.13)			
Left Rotation	1.28 (0.98)	1.21 (1.08)			
Extension	1.57 (1.32)	1.89 (1.47)			
Flexion	1.43 (0.96)	1.00 (0.75)			
OPE*sagittal*			0.057	<0.001 *	0.395
Right Rotation	1.82 (0.84)	1.71 (0.87)			
Left Rotation	2.07 (1.31)	2.00 (1.34)			
OPE*horizontal*					
Extension	1.98 (1.28)	1.38 (0.91)			
Flexion	1.15 (0.93)	0.99 (0.82)			
OPE*frontal*			0.151	<0.001 *	0.051
Right Rotation	2.39 (1.94)	2.63 (2.24)			
Left Rotation	2.50 (1.67)	2.07 (1.69)			
Extension	1.75 (1.22)	0.87 (0.65)			
Flexion	0.77 (0.55)	0.90 (0.63)			
OPE*3D*			0.548	0.031 *	0.139
Right Rotation	1.16 (1.01)	1.63 (1.40)			
Left Rotation	1.56 (1.36)	1.56 (1.35)			
Extension	1.61 (1.34)	1.91 (1.48)			
Flexion	1.39 (0.94)	0.96 (0.73)			
**(b)**					
Direction		OPE*int-component*	OPE*sagittal/horizontal*	OPE*frontal*	OPE*3D*
Left Rotation vs. Right Rotation		0.847	0.056	0.281	0.382
Extension vs. Right Rotation		0.002 *	0.670	<0.001 *	0.069
Flexion vs. Right Rotation		0.654	<0.001 *	<0.001 *	0.287
Extension vs. Left Rotation		0.037	0.186	<0.001 *	0.458
Flexion vs. Left Rotation		0.854	<0.001 *	<0.001 *	0.088
Flexion vs. Extension		0.019 *	<0.001 *	0.001 *	0.008 *

* *p* < 0.05.

**Table 3:** (a) Mean (standard deviation, SD) in degrees (°) and ANOVA results for JPE outcomes. (b) Student’s *t*-test post hoc on contrasts between pairs for JPE*intended* and JPE*3D* for the “Direction” factor. (c) Student’s *t*-test results on contrasts between pairs for JPE*sagittal*, JPE*horizontal*, and JPE*frontal* for “Operator × Direction” interaction factor. Coefficients of variation are presented only for JPE*3D*. The P-value was set at *p* < 0.05.

**(a)**							
Direction	Operator 1	Coefficient of Variation	Operator 2	Coefficient of Variation	*p*-Value (Operator)	*p*-Value (Direction)	*p*-Value (Operator × Direction)
JPE*int-component*					0.112	<0.001 *	0.742
Right Rotation	5.97 (5.29)		4.81 (5.26)				
Left Rotation	5.27 (4.71)		4.80 (4.32)				
Extension	2.04 (4.08)		1.55 (3.79)				
Flexion	0.61 (4.55)		0.48 (4.15)				
JPE*sagittal*					0.660	<0.001 *	<0.001 *
Right Rotation	−1.68 (2.31)		−1.03 (2.61)				
Left Rotation	0.27 (2.25)		1.00 (2.45)				
JPE*horizontal*							
Extension	1.28 (1.67)		0.09 (1.76)				
Flexion	−0.81 (1.59)		−0.68 (1.82)				
JPE*frontal*					<0.001 *	<0.001 *	0.004 *
Right Rotation	−0.35 (1.51)		−0.03 (1.79)				
Left Rotation	0.02 (1.47)		0.28 (1.65)				
Extension	−0.88 (1.21)		0.80 (1.13)				
Flexion	−1.00 (0.99)		−0.65 (1.04)				
JPE*3D*					0.106	<0.001 *	0.988
Right Rotation	6.87 (3.89)	0.57	6.34 (3.11)	0.49			
Left Rotation	5.97 (2.86)	0.48	5.59 (2.22)	0.40			
Extension	4.34 (1.78)	0.41	3.84 (1.83)	0.48			
Flexion	3.92 (2.01)	0.51	3.61 (1.68)	0.46			
**(b)**							
Direction			JPE*int-component*			JPE*3D*	
Left Rotation vs. Right Rotation			0.544			0.022	
Extension vs. Right Rotation			<0.001 *			<0.001 *	
Flexion vs. Right Rotation			<0.001 *			<0.001 *	
Extension vs. Left Rotation			<0.001 *			<0.001 *	
Flexion vs. Left Rotation			<0.001 *			<0.001 *	
Flexion vs. Extension			0.010			0.277	
**(c)**							
			JPE*sagittal/horizontal*			JPE*frontal*	
Operator 1	Left Rotation vs. Right Rotation		<0.001 *			0.396	
	Extension vs. Right Rotation		<0.001 *			0.052	
	Flexion vs. Right Rotation		0.027			0.035 *	
	Extension vs. Left Rotation		0.013 *			0.017 *	
	Flexion vs. Left Rotation		0.003 *			<0.001 *	
	Flexion vs. Extension		<0.001 *			0.620	
Operator 2	Left Rotation vs. Right Rotation		<0.001 *			0.502	
	Extension vs. Right Rotation		0.026 *			0.036 *	
	Flexion vs. Right Rotation		0.491			0.086	
	Extension vs. Left Rotation		0.047 *			0.088	
	Flexion vs. Left Rotation		<0.001 *			0.005 *	
	Flexion vs. Extension		0.106			<0.001 *	
Right Rotation	Operator 1 vs. Operator 2		0.004 *			0.175	
Left Rotation	Operator 1 vs. Operator 2		0.017 *			0.137	
Extension	Operator 1 vs. Operator 2		<0.001 *			<0.001 *	
Flexion	Operator 1 vs. Operator 2		0.541			0.030 *	

* *p* < 0.05.

## Data Availability

The data presented in this study are available on request from the corresponding author.

## Supplementary Materials

The following supporting information can be downloaded at: https://www.mdpi.com/article/10.3390/brainsci13040604/s1, Figure S1: Sketch of the trial design; Table S1: Example of trial sequence randomisation; Table S2: Reference values for clinical research.
