# Supplementary material for: Cervical Proprioception Assessed through Targeted Head Repositioning: Validation of a Clinical Test Based on Optoelectronic Measures
Source: Brain Sci. 2023 Apr 2;13(4):604. doi: 10.3390/brainsci13040604 (PMC10136693; doi:10.3390/brainsci13040604)
Supplement: Supplementary file 1 [file brainsci-13-00604-s001.zip › brainsci-2269172-supplementary.pdf]

SUPPLEMENTARY MATERIAL FOR

Cervical Proprioception Assessed through Targeted Head Repositioning: Validation of a Clinical Test Based on Optoelectronic Measures

Brain Sci. 2023

Valeria Cerina[1], Luigi Tesio[1*], Chiara Malloggi[1], Viviana Rota[1], Antonio Caronni [1,2], and Stefano Scarano[1,2]

1. Istituto Auxologico Italiano, IRCCS, Department of Neurorehabilitation Sciences, Ospedale San Luca, Milan, Italy

2. Department of Biomedical Sciences for Health, University of Milan, Milan, Italy

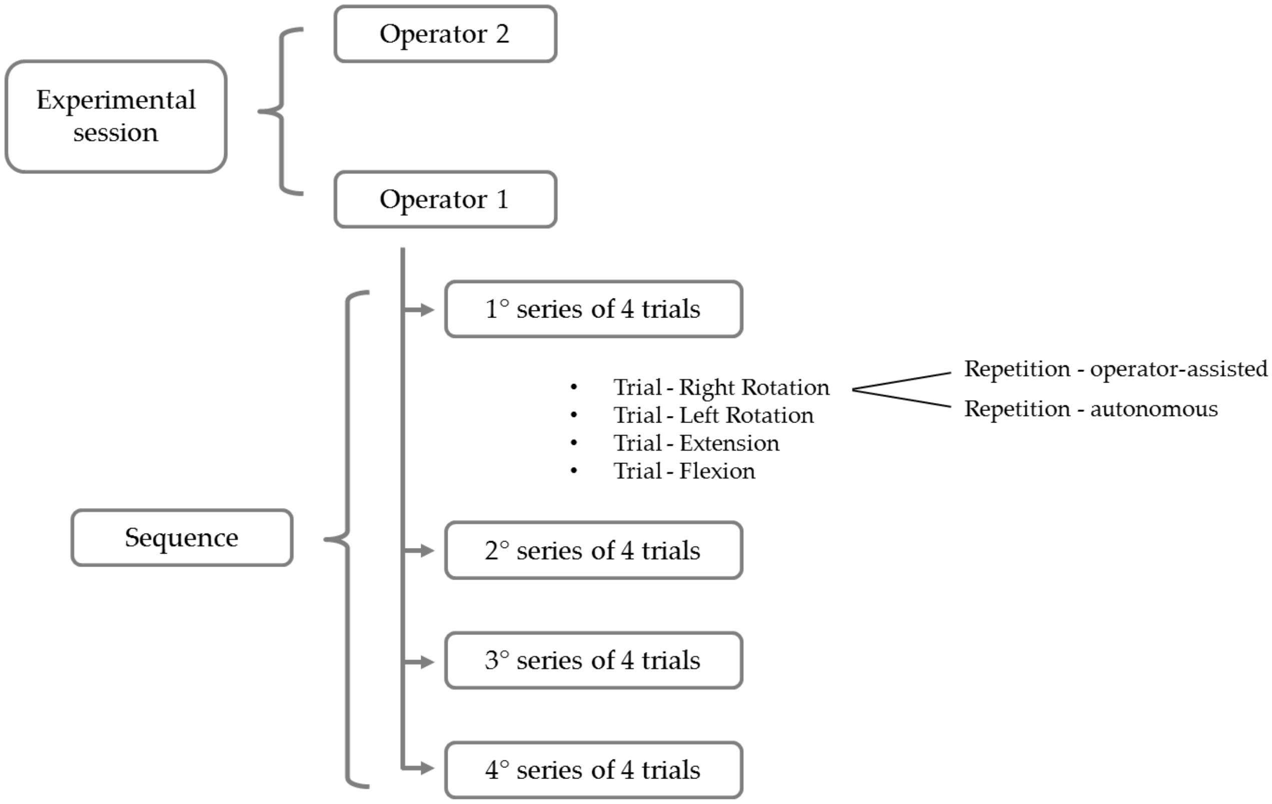

Supplementary Figure S1: Sketch of the experimental design. A "trial" included both one assisted and one autonomous "repetition" of the same movement into one of four directions. Each of the two operators conducted one of the two "sequences" (i.e., 16 trials each). The "experimental session" included 32 trials (i.e., 32 assisted and 32 autonomous repetitions – see text for details), i.e., 64 repetitions overall. The directions of trials (i.e., right rotation, left rotation, extension, and flexion) were quasi-randomised across patients and operators.

Supplementary Table S1. Example of trial sequence quasi-randomisation. Four ***SEQUENCES*** (16 ***TRIALS*** each) associated with the two operators for Participant 1 and Participant 2 are reported. E* represents the starting ***TRIAL*** raffled off at the beginning of the study. The default series was F, RR, E, LR (i.e., Flexion; Right Rotation; Extension, and Left Rotation, respectively). The starting Trials of the following three series of four trials follow in the loop of the default series. In this example, for Participant 1, they will be LR, F, and RR (underlined in the Table). The Starting Trial for the second participant and the Starting Trial for the sequence associated with the second operator for Participant 1 follow the default series. In this example, they will be LR (bolded in the Table).

**Participant 1**	**(OPERATOR 1)**				**(OPERATOR 2)**			
First series of four Trials	E*	LR	F	RR	**LR**	F	RR	E
Second series of four Trials	<u>LR</u>	F	RR	E	F	RR	E	LR
Third series of four Trials	<u>F</u>	RR	E	LR	RR	E	LR	F
Fourth series of four Trials	<u>RR</u>	E	LR	F	E	LR	F	RR
**Participant 2**	**(OPERATOR 2)**				**(OPERATOR 1)**			
First series of four Trials	**LR**	F	RR	E	F	RR	E	LR
Second series of four Trials	F	RR	E	LR	RR	E	LR	F
Third series of four Trials	RR	E	LR	F	E	LR	F	RR
Fourth series of four Trials	E	LR	F	RR	LR	F	RR	E

Supplementary Table S2. Reference values for the first sequence of 16 trials for the 26 participants (assessed by one operator, only). Mean, standard deviation, median, 90th percentile, 95th percentile, the standard error for planar JPEs (i.e., JPE*int-component*, JPE*sagittal*, JPE*horizontal*, and JPE*frontal*, °) and three-dimensional Joint Position Error (JPE*3D*, °) for the four *trial directions* and the whole *sequence* (rightmost column). Only the first sequence performed with the first operator was considered (i.e., the first sixteen trials for each participant). Each cell of the first four columns summarises 104 trials. Each cell of the Overall column summarises 416 trials.

**JPE*int-component***	**Right Rotation**	**Left Rotation**	**Extension**	**Flexion**
mean	5.47	4.77	1.82	0.43
standard deviation	4.96	4.23	3.67	4.26
median	5.72	4.73	1.47	-0.24
90th percentile	11.48	10.12	6.82	5.23
95th percentile	13.04	10.71	8.06	8.06
standard error	0.49	0.41	0.36	0.42
**JPE*sagittal***				
mean	-1.22	0.70		
standard deviation	2.26	2.61		
median	-1.24	0.70		
90th percentile	1.49	3.35		
95th percentile	1.82	4.13		
standard error	0.22	0.26		
**JPE*horizontal***				
mean			0.73	-0.68
standard deviation			2.01	1.65
median			0.89	-0.61
90th percentile			2.99	1.22
95th percentile			3.89	1.79
standard error			0.20	0.16
**JPE*frontal***				
mean	0.38	0.16	-0.12	-0.84
standard deviation	1.68	1.53	1.27	1.03
median	0.72	0.06	-0.11	-0.86
90th percentile	1.79	1.73	1.51	0.52
95th percentile	2.34	2.51	2.14	0.88
standard error	0.16	0.15	0.12	0.10
**JPE*3D***				
mean	6.68	5.58	4.02	3.63
standard deviation	3.92	3.10	2.23	2.51
median	6.19	5.05	3.58	3.18
90th percentile	12.25	10.37	7.36	6.77
95th percentile	13.28	11.44	8.44	8.46
standard error	0.38	0.30	0.22	0.25
